# Parathyroid Carcinoma: An Up-to-Date Retrospective Multicentric Analysis

**DOI:** 10.1155/2020/7048185

**Published:** 2020-03-06

**Authors:** Francesco Quaglino, Luca Manfrino, Luca Cestino, Massimo Giusti, Enrico Mazza, Alessandro Piovesan, Nicola Palestini, Corrado Lauro, Elena Castellano

**Affiliations:** ^1^Department of General Surgery, “Maria Vittoria” Hospital ASL Città di Torino, Turin 10144, Italy; ^2^Department of Internal Medicine, “San Giovanni Bosco” Hospital ASL Città di Torino, Turin 10154, Italy; ^3^Department of Endocrinology, “Maria Vittoria” Hospital ASL Città di Torino, Turin 10144, Italy; ^4^Department of Endocrinology, A. O. U. Città della Salute della Scienza di Torino, Turin 10126, Italy; ^5^Thyroid and Parathyroid Surgery, Candiolo Cancer Institute I. R. C. C. S., Candiolo 10060, Italy; ^6^Department of General Surgery, “Santa Croce e Carle” Hospital, Cuneo 12100, Italy; ^7^Department of Endocrinology, Diabetes and Metabolism, “Santa Croce e Carle” Hospital, Cuneo 12100, Italy

## Abstract

Parathyroid carcinoma (PC) is a rare disease responsible for about 1% of primary hyperparathyroidism (PHPT) cases. PC usually has an indolent course, tough to differentiate from the benign causes of PHPT, and the only certain diagnosis is histologic. The gold standard surgical treatment is the en bloc resection associated with the homolateral thyroid loboistmectomy. The aim of this study was to underline the main differences between PC and benign PHPT, along with gathering epidemiological knowledge relative to PC in our region. Data from the regional cancer network (Rete Oncologica del Piemonte e della Valle d'Aosta) since 2007 have been reported, including 21 patients from three hospitals (AO S. Croce e Carle of Cuneo, AOU Città della Salute of Turin, and ASL Città di Torino). The incidence of the disease, gender, age at time of diagnosis, presence of renal and bone symptoms, serum calcium and PTH levels, surgical technique performed, and percentage of recurrence were analysed. PC data were than compared with a series of patients affected by benign PHPT, referred to ASL Città di Torino, Maria Vittoria Hospital, from 2007 to 2019. A PC incidence of 0.05 cases per 100,000 inhabitants was found in our region. Benign forms occurred more frequently in females (*p*=0.0002), while PC equally occurred in males and females and affected younger patients (*p*=0.026). Serum calcium and PTH levels were significantly higher in PC patients; accordingly, typical PHPT symptoms were more frequently reported in PC than in benign PHPT. In the PC group, the en bloc resection shows a 13 times lower risk for relapse compared with all the other surgical techniques. PC is equally gender distributed, and the average patients' age is in the fifth decade of life. It is usually functioning, with greater biochemical activity and multiple symptoms. A not-radical surgical resection is associated with a higher recurrence rate. A meticulous presurgical evaluation of PHPT patients showing PC's evocative features is mandatory to obtain a complete disease extirpation.

## 1. Introduction

Parathyroid carcinoma (PC) is a rare disease responsible for about 1% of primary hyperparathyroidism (PHPT) cases and represents 0.005% of all tumours [1, 2]. PC is usually sporadic, but it can be associated with genetic syndromes [3].

Its aetiology is unknown, but a correlation with radiation exposure and with secondary and tertiary hyperparathyroidism associated with renal failure has been reported [4].

Parathyroid carcinomas usually have an indolent course [5, 6], and given the extreme clinical difficulty in differentiating them from the benign causes of primary hyperparathyroidism, to date, the only certain diagnosis is histologic.

An important characteristic of PC is the recurrence rate which stands at a value of 50% [7]. Furthermore, relapse seems to be related to the surgical technique used to remove the tumour [8]. The gold standard surgical treatment is the en bloc resection associated with the homolateral thyroid loboistmectomy [9, 10].

However, this technique is not always performed because physicians often underestimate the possibility of the lesion's malignancy.

The association of prophylactic central neck dissection with the en bloc resection seems to be controversial. Lymph node involvement is documented in the literature in 6.5% of patients [11], and its prognostic role is not clarified. The studies on this topic show different recommendations: some suggest a central neck dissection while others do not [12–18].

Therefore, the aim of this study was to highlight the main clinical and biochemical differences between PC and benign causes of PHPT. A further purpose was to gather epidemiological knowledge relative to PC in our region in the interest to support the regional cancer network “Rete Oncologica del Piemonte e della Valle d'Aosta” [19].

Moreover, the en bloc resection was compared with other less radical surgical techniques in order to look for consequences on relapse probability.

## 2. Materials and Methods

30 patients were diagnosed with PC during the last 12 years (from January 2007 to July 2019), and most of their data were inserted in the regional cancer network. Complete information was available only for 21 patients which were included in the study.

The patients were treated in three different hospitals: AOU Città della Salute e della Scienza di Torino, ASL City of Turin (Maria Vittoria Hospital), and AO Santa Croce e Carle of Cuneo.

All patients underwent parathyroid resection surgery with subsequent histologic diagnosis.

The pathological criteria considered suggestive of PC were [20, 21] capsule and surrounding structures' invasion, vascular invasion, and presence of metastases.

None of the patients examined had lymph node involvement and/or distant metastasis.

A comparison was then performed with a series of 92 patients with a clinical evidence of PHPT without malignancy characteristics diagnosed in a period from January 2007 to July 2019 at Maria Vittoria hospital (ASL City of Turin). Among these 92 subjects, 50 underwent surgery in our surgical service and had a histologic diagnosis confirmation of single parathyroid adenoma. PHPT patients have been addressed to surgical treatment if symptomatic or asymptomatic, meeting the surgical criteria reported by the latest international guidelines [22].

Regarding PC, the following characteristics were examined: sex, age at the time of diagnosis, symptoms, serum calcium level, PTH (normal values, 16–75 pg/ml [10]), side of the lesion, size of the tumour, surgical technique used, and disease recurrence.

The comparison with the population affected by benign PHPT concerns the following characteristics: sex, age, symptoms, serum calcium level, and PTH.

Renal (nephrolithiasis, nephrocalcinosis, and renal insufficiency) and bone (osteoporosis, fractures, brown tumours, and cystic fibrous osteitis) involvement were reported.

## 3. Statistical Analysis

Variables were preliminarily tested for normal distribution with the Shapiro–Wilk test, and data were expressed as mean ± SD when normally distributed and as median and interquartile range (IQR) when not normally distributed. The chi-squared test was used for the categorical variables, except when the contingency tables presented values ≤5 where the Fisher test was employed. For noncategorical variables, the Shapiro–Wilk test was used to verify the normal distribution of the sample, in which case the data were compared with the Student's *t*-test and expressed as mean ± standard deviation.

In the event of a not normal distribution, the data were compared with the Wilcoxon rank-sum test and described as median and interquartile range. The significance levels considered for two-sided *P* values were 0.05 in all tests.

## 4. Results and Discussion

### 4.1. Results

Table 1 summarizes the characteristics of the whole series of PC patients, while Table 2 shows the comparison with patients with benign PHPT.

First of all, the incidence in our region resulted to be of 0.05 cases per 100,000 inhabitants.

As far as sex and age are concerned, benign forms seem to occur more frequently in the female sex (*P*=0.0002) and in older persons, while PC affects younger patients (*P*=0.026) without substantial differences in incidence between the two genders.

19 out of the 21 (90.5%) PC patients presented symptoms at the time of the diagnosis, while only 40 out of 92 (43.5%) patients suffering from benign forms on PHPT were symptomatic.

It is interesting to note that, in malignant tumours, both kidney and bone symptoms are reported.

Also taking singularly the bone and then the renal symptoms, a significant difference between the two populations can be noted (*P*=0.0002 and *P*=0.0001).

Serum calcium and PTH values resulted significantly higher in the PC group.

The relationship between the type of surgical technique used and the percentage of relapse is summarized in Table 3.

In 4 patients, the mutation of CDC73 was investigated and none of them displayed abnormalities.

The gold standard surgical technique (en bloc resection) was used only on 10 (47.6%) patients, none of whom developed recurrence. The other 11 (52.4%) were treated with less radical resections, and in 6 of them, the tumour relapsed (54.5%).

The disease recurrence was significantly lower in the patients undergoing en bloc resection compared with all the others techniques (*P*=0.0152; OR 13).

The univariate linear regression model (Table 4), used to establish the level of correlation between the PC and presence of symptoms and serum calcium and PTH levels, showed a positive correlation with all the examined parameters, being moderate-strong only for PTH (*R*=0.689 and *R*^2^=0.475).

### 4.2. Discussion

Parathyroid carcinoma is a rare tumour, and it is difficult to assert a clinical diagnosis based only on symptoms and biochemical characteristics.

In the literature, there is a lack of studies with a relevant number of patients because of the epidemiological features of the disease.

In our study, the main characteristics of PC in our region's population were defined and compared with benign forms of PHPT in order to highlight the main differences and help the diagnostic process.

A study of RARECAREnet Project [2] indicates an incidence of PC in Europe of 0.03/100,000.

Some features were found to be helpful with the diagnosis of PC. Benign PHPT showed a greater incidence in females, while PC's incidence is similar in the two genders [13, 23, 24].

This hallmark was confirmed in our study. Moreover, the mean age of PC presentation is usually lower than benign PHPT [10]. This finding has been confirmed in our study, even if a little older compared with the statistics found in the literature [4].

Most of the PCs are functioning [13, 25], causing a great PTH elevation and multiple symptoms. The main districts interested are the bones and kidneys. In our PC population, over 90% patients presented symptoms and more than half of them had a multiple district involvement.

On the other hand, in the benign PHPT population, just under half patients presented symptoms and mostly with only one district affected.

Serum calcium and PTH levels in PC are usually higher than those in benign PHPT [3, 26–28]. This finding was confirmed in our study; in particular, PTH shows 3 or more times higher levels in PC, significantly higher than the values reported in our benign PHPT cases.

According to the results obtained, the en bloc resection appears to be associated with a lower risk of recurrence, while a less radical resection with a 13 times higher relapse risk.

An additional interesting feature concerning PC is the relation with germline mutations of CDC73, a gene that encodes a protein known as parafibromin [10]; anomalies in this gene associate with higher probability to suffer PC-isolated or in the context of hyperparathyroidism-jaw tumour syndrome or familial-isolated primary hyperparathyroidism [29].

Unfortunately, as this evaluation was available only in 4 patients, no statistical analysis was performed.

In this study, all the three regional hub centres for PC were included; for this reason, it is likely that all the PC regional cases were included. Therefore, our PC rate may be affected by selection bias.

The main limitation of the study is that the benign PHPT patients were selected only from one hospital, so they may not be totally representative of the regional population.

## 5. Conclusion

Investigating all the clinical features that could differentiate PC from benign PHPT is crucial to ensure a correct surgical approach, reducing the risk of recurrence and the necessity of reoperation, which is burdened by a higher frequency of surgery-related complications.

Furthermore, it is difficult to eradicate the disease completely after recurrence, forcing the patient to live with a hard-to-treat severe hypercalcemia syndrome [30].

## Figures and Tables

**Table 1:** Characteristics and treatment of patients affected by PC.

Patient no.	Sex	Age	Symptoms	Ca (mg/dl)	PTH (pg/ml)	Side	Size (cm)	Surgical technique	Relapse
1	F	60	B, K	13	1195	L	5.7	En bloc	No
2	F	70	B, K	12.8	1170	R	na	Other	Yes
3	M	37	No	13.4	602	R	na	En bloc	No
4	F	82	K	9.6	501	L	2	En bloc	No
5	F	38	K	12.6	353	R	4	Other	No
6	F	76	No	13.3	675	L	2	Other	Yes
7	F	57	B, K	12.6	1497	R	4.5	Other	No
8	M	59	B	13	667	R	6	Other	No
9	M	49	B, K	15.4	1140	L	5	Other	No
10	M	50	B, K	15	900	R	na	Other	Yes
11	M	61	B, K	12	464	L	2	En bloc	No
12	M	82	B	12.8	427	na	1.5	Other	Yes
13	M	38	B, K	8, 3	692	R	5	En bloc	No
14	F	67	B, K	13	700	R	1.5	En bloc	No
15	F	32	B	10	758	na	4	Other	No
16	F	76	B, K	13.6	1916	L	3.2	Other	Yes
17	M	33	B, K	16.9	3050	R	2.5	En bloc	No
18	M	77	B	11	915	R	3	En bloc	No
19	M	67	K	12.8	683	L	4	En bloc	No
20	F	45	B	13.6	908	R	4.9	En bloc	No
21	M	59	B, K	10.5	920	L	5.5	Other	Yes

F = female, M = male, B = bone involvement, K = kidney involvement, L = left, R = right, and NA = not available.

**Table 2:** Comparison between PC and benign PHPT populations.

Variable	PC	Benign PHPT	*P* value
No. of cases	21	92	—
Sex, M/F	11/10	16/76	0.0002
Age			
Median (IQR)	59 (27.5)	68 (14.5)	0.026
Mean ± SD	57.96 ± 16.61	65.98 ± 12.26	—
Patient with symptoms	19	40	0.0001
Bone involvement	16	27	0.0002
Kidney involvement	14	16	0.0001
Ca (mg/dl)			
Mean ± SD	12.63 ± 1.97	10.69 ± 1.14	10^–9^
PTH (pg/ml)			
Median (IQR)	758 (473)	137.5 (100.5)	10^–6^
Side, R/L	11/8	—	—
Size (cm)			
Mean	3.63	—	—

Ca = calcium, R = right, L = left, IQR = interquartile range, and SD = standard deviation.

**Table 3:** Comparison between the en bloc resection and less radical resections.

	En bloc resection	Other resection	*P* value	OR
No. of patients	10	11	0.0152	13
Relapse	0	6	—	—
No relapse	10	5	—	—

**Table 4:** Univariate linear regression.

Variable	*R*	*R* ^2^	*P* value
Symptoms	0.369	0.136	<0.0001
Calcium	0.493	0.243	0.005
PTH	0.689	0.475	<0.0001

## Data Availability

The patient data used to support the findings of this study are available from the corresponding author upon request.
